# Effect of alverine citrate plus simethicone in colonoscopy: a randomized controlled trial

**DOI:** 10.1038/s41598-024-62922-2

**Published:** 2024-05-27

**Authors:** Chumpon Wilasrusmee, Jakrapan Jirasiritham, Chairat Supsamutchai, Puvee Punmeechao, Napaphat Poprom

**Affiliations:** 1 Department of Surgery, Faculty of Medicine, Ramathibodi Hospital, Mahidol University, Salaya, Thailand; 2 Faculty of Public Health, Chiang Mai University, 239, Huay Kaew Road, Muang District, Chiang Mai, 50200 Thailand

**Keywords:** Alverine citrate, Simethicone, Colonoscopy, Antispasmodic drug, Gastroenterology, Medical research

## Abstract

Colonoscopy is the standard procedure for screening, and surveillance of colorectal cancer, including the treatment for colonic lesions. Colonic spasm is an important problem from colonoscopy that affects both surgeons and patients. The spasm also might be the cause of longer cecal intubation time, difficulty of the procedure, and increased pain. Previous reports indicated that antispasmodic agents can decrease such symptoms. Therefore, we conducted this study to investigate the cecal intubation time of antispasmodic agents. A single blinded randomized controlled trial was conducted from 01/11/2020 to 31/08/2021. One hundred four patients were allocated to antispasmodic agent group and control group, in 1:1 ratio. The efficacy of median (range) cecal intubation time showed similar results of 5 (2, 14) and 5 (2, 15) minutes with no statistically significant difference. The mean scores of all domains i.e., pain, spasm, cleanliness, and difficulty were better in the antispasmodic agent group about 2.6 (1.4), 1.8 (0.8), 2.4 (0.9), and 2.0 (0.9), respectively, than control group but there were spasm and cleanliness showed statistically significant difference. Moreover, the satisfaction scores showed better efficacy in decreased spasm, decreased difficulty, and increased cleanliness than control group. Prescribing of antispasmodic drugs before colonoscopy might be the choice of treatment for the patients. The antispasmodic drugs will be beneficial to both of the patient and the doctor.

## Introduction

Colonoscopy is the standard procedure for screening, and surveillance of colorectal cancer, including the treatment for colonic lesions^1^. Therefore, screening colonoscopy according to the national guidelines and Clinical Practice Guideline for Colorectal Cancer of Thailand should be performed in patients aged from 45 to 75 years old^2–4^. The rate of colonoscopy with or without patient preference was increased ranging from 17 to 25.7%^3,5–10^. Moreover, colonoscopy incorporated with high accuracy from 82 to 93% to detect colorectal cancer^11^. However, the rate of colonoscopy has increased due to easy setting with safer but those might affect the cost and the waiting list of the patients^11,12^.

Furthermore, colonic spasm is an important problem from colonoscopy and has an impact on the patients and the physicians as well^13,14^. It leads to increased cecal intubation times or insertion times, difficulty of colonoscopy, and causes pain and discomfort for the patients. Although, those symptoms may be temporarily expressed they occur frequently with effect during and post of colonoscopy in both physicians and patients^13^.

Antispasmodic agents have been reported in both directions in terms of beneficial to relieving colonic spasm symptoms during colonoscopy^13,15–21^. Some studies indicated that antispasmodic agents can reduce pain because those agents are associated with the mechanism of contractile pathway inhibition in the gut wall. Only one randomized control trial (RCT) concluded that the scores of cleanliness in antispasmodic agent group higher than control group^13^.

The evidences of previous meta-analyses^22–24^ suggested and proved the efficacy of antispasmodic agents in the treatment of irritable bowel syndrome (IBS). However, antispasmodic agents are available differently in different countries. Additionally, the recommendation of effectiveness from Gastroenterology could not concluded because of insufficient information from many countries with inconsistent results in both IBS and colonoscopy patients^22,25,26^.

Therefore, we conducted a randomized controlled trial to investigate the efficacy regarding cecal intubation time. The secondary outcomes explored the score of pain, spasm, difficulty, and increased cleanliness of antispasmodic agents (alverine citrate plus simethicone) in colonoscopy in Thailand.

## Materials and methods

The primary objective of this study was to compare the cecal intubation time between antispasmodic agent group and non-received antispasmodic group. Secondary objective was to explore the scores in term of pain, spasm, difficulty, and cleanliness between antispasmodic agent group and non-received antispasmodic group.

The study design and method were conducted following the consolidated standards of reporting trials (CONSORT) guidelines^27^ and registered in the Thai Clinical Trials Registry on 19/02/2021 with number: TCTR20210219008*.* The study was confirmed that all participants were signed informed consent according to the Declaration of Helsinki.

### Study design

A single blinded randomized controlled trial was conducted from 01/11/2020 to 31/08/2021. The study protocol was approved and consented by the ethical committee of Ramathibodi Hospital (#COA. MURA2020/1892). All patients who met our inclusion criteria received the study information and signed consent forms before participated.

The satisfied patients were randomized to either receive antispasmodic agents or non-received antispasmodic, in 1:1 ratio with the physician single blinded.

### Patient selection

Patients were eligible in the study if they met the criteria: All adult patients aged 17–80 years and received colonoscopy.

Patients were excluded from the study for several reasons including those with a history of bowel obstruction, colorectal cancer, or colorectal surgery. Additionally, patients displaying signs or symptoms of glaucoma, obstructive uropathy, acute lower gastrointestinal bleeding, renal failure, or heart failure were also excluded. Furthermore, patients who had history or recorded of alverine citrate and simethicone allergies were not included in the study.

### Intervention and outcomes

Colonoscopy is capable of examining the entire colon and terminal ileum with complete bowel preparation is necessary. The sedation of colonoscopy procedure should be a concern for duration and discomfort control^28^.

The patients who were allocated to the intervention group were prescribed antispasmodic drugs 5 days (3 times a day before meals) before the date of colonoscopy. Both of the patient groups received 90 ml of sodium phosphate 1 day before colonoscopy and 133 ml of sodium phosphate 30 min with the suggestion of control diet consumption before colonoscopy for bowel preparation.

The primary outcome of the cecal intubation time was defined as the time from the colonoscopy tip insertion into the anal verge until reaching the cecal base^13^. The secondary outcomes included pain, spasms, difficulty, and increased cleanliness.

### Data collection

Pain was defined as pain scores at the end of the operation, which were graded to 0; no pain, 1; mild, 2; moderate, 3; severe, and 4; very severe and using a visual analog scale 1–10 (VAS). Spasm was defined as intestinal spasm as luminal narrowing of one-third or greater of the circumference of the lumen and interfere with colonoscopy insertion^29,30^, which was graded to 0; no spasm at all, 1; spasm that did not interfere with colonoscopy insertion, 2; spasm strong enough to interfere with colonoscopy insertion but not sufficient to prevent completion of the colonoscopy, 3; spasm severe enough to prevent completion of the colonoscopy, and/or to push the colonoscopy backward out of position, and with intermittent air insufflation needed throughout the examination, and 4; spasm severe enough to prevent completion of the colonoscopy, and/or to push the colonoscopy backward out of position, and with continual air insufflation needed throughout the examination. The difficulty was defined as the scores of difficulty of colonoscopy insertion and grade to 0; very easy, 1; easy, 2; not easy but not difficult, 3; difficult, and 4; very difficult. The cleanliness was defined as the scores of the circumference of colon visualization with a grade of 0; hard stool, more than 50% of the circumference of the colon cannot be visualized, 1; smooth stool, more than 50% of the circumference of the colon cannot be visualized, 2; smooth stool, between 25 and 50% of the circumference of colon cannot be visualized, 3; smooth stool, less than 25% of the circumference of colon cannot be visualized, and 4; no stool^13^.

### Statistical analysis

The intention to treat (ITT) was applied in this study. The characteristic information of the dichotomous variables was reported by the number with the percentage using chi-square test. The continuous variables were reported by the mean with standard deviation using student t-test. Both analyses were held on the assumption if there was not met the assumption, the Fisher’s exact test and Mann–Whitney test were applied, respectively.

The outcomes of the study were reported in mean and standard deviation using a student t-test with normal distribution. In contrast, if there was no normal distribution, the median with range was reported using Mann–Whitney test.

The sample size estimation of this study applied the formula of test comparing two independent means using mean with standard deviation of cecal intubation time from previous evidence^13^ among intervention and control groups of 6.20 ± 3.24 and 7.48 ± 3.45 min, respectively, with alpha error was 0.05 and beta error was 0.2. The total sample size with the rate of 10% loss follow-up should be 240 patients. However, the COVID-19 pandemic affected our study greatly. Therefore, we could not collect the data reached to the target sample sizes. The STATA program version 17 was used for data analysis.

## Results

### Characteristics information of eligible studies

**Figure 1:**
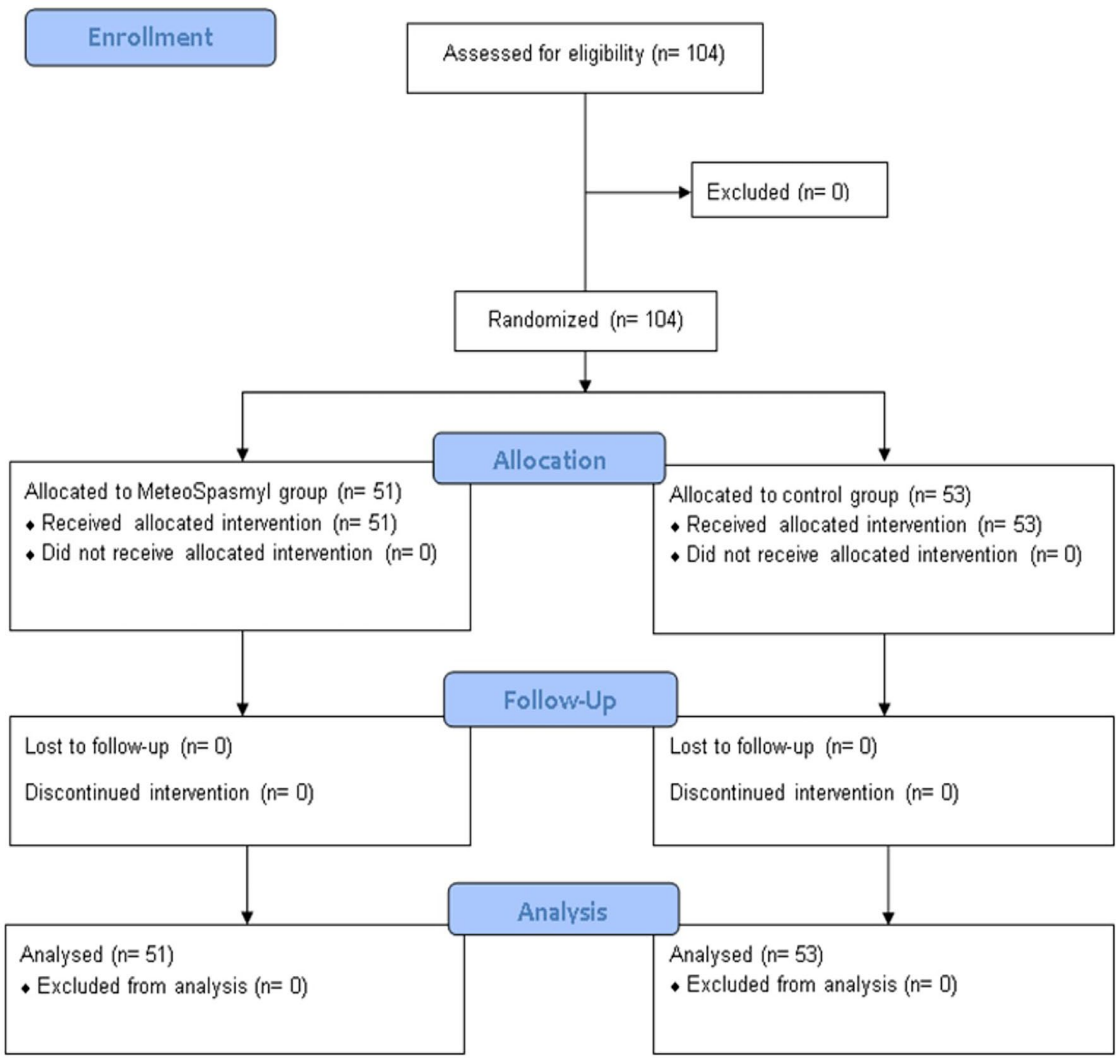
Consort flow diagram.

One hundred and four patients met our inclusion criteria and entered the study. Among those patients, there were no patients excluded from the study for any reason. From 104 selected patients, 51 patients were randomly allocated to antispasmodic agent group and 53 patients were allocated control group or non-received antispasmodic agent, see Fig. 1.

Baseline characteristics of ITT patients showed balance between two groups of intervention. The mean age of antispasmodic agent and control group were 63.3 (12.2) and 58.0 (15.4) years, respectively. The percentage of male was nearly similar to 47.1% and 45.3% in antispasmodic agent and control groups, respectively. There was no difference in all variables, including BMI, smoking status, abdominal pelvic operation history, laxative usage status, colonoscopy conditions, and final diagnosis, see Table 1.

**Table 1:** Characteristic information of the patients.

Variables	Antispasmodic group (n = 51)	Control group (n = 53)	*p* value
Gender, N (%)			0.856
Male	24 (47.1)	24 (45.3)	
Female	27 (52.9)	29 (54.7)	
Age (year), mean (SD)	63.3 (12.2)	58.0 (15.4)	0.051
Weight (kilogram), mean (SD)	64.7 (11.6)	62.9 (10.3)	0.380
Height (centimeter), mean (SD)	161.9 (8.9)	161.1 (7.9)	0.635
BMI (kg/m^2^), mean (SD)	24.7 (3.9)	24.2 (3.2)	0.462
Smoking, N (%)			0.716
Yes	3 (5.9)	5 (9.4)	
No	48 (94.1)	48 (90.6)	
ABD pelvic operation history, N (%)			0.861
Yes	23 (45.1)	23 (43.4)	
No	28 (54.9)	30 (56.6)	
Laxative usage, N (%)			0.867
Yes	29 (56.9)	31 (58.5)	
No	22 (43.1)	22 (41.5)	
Colonoscopy conditions, N (%)			0.134
ABD pain	4 (7.8)	5 (9.4)	
Cancer and cancer suspicious	16 (31.4)	17 (32.1)	
Bowel syndrome	0 (0.0)	4 (7.5)	
Constipation	28 (54.9)	20 (37.7)	
Rectal bleeding	3 (5.9)	7 (13.2)	
Diagnosis, N (%)			0.261
Hemorrhoid	15 (29.4)	24 (45.3)	
Cancer	1 (1.9)	2 (3.8)	
Polyp	26 (50.9)	15 (28.3)	
Colitis	1 (1.9)	1 (1.9)	
Diverticulitis	5 (9.8)	6 (11.3)	
Normal	3 (5.9)	5 (9.4)	

ABD, abdominal; BMI, body mass index; N, number; SD, standard deviation.

### Primary efficacy outcome

**Figure 2:**
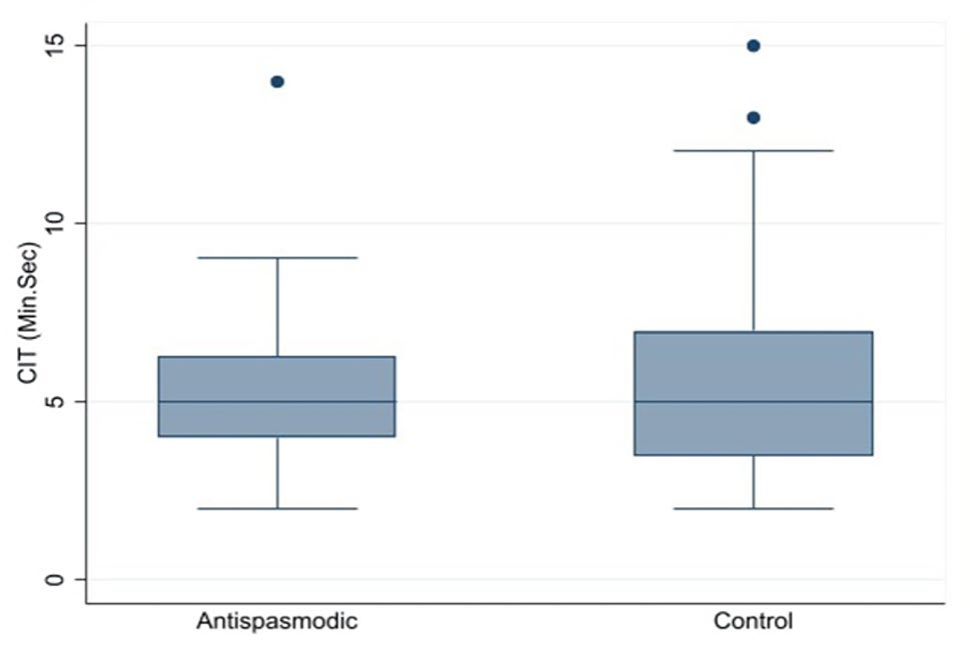
Mean of cecal intubation time among 2 interventions.

**Figure 3:**
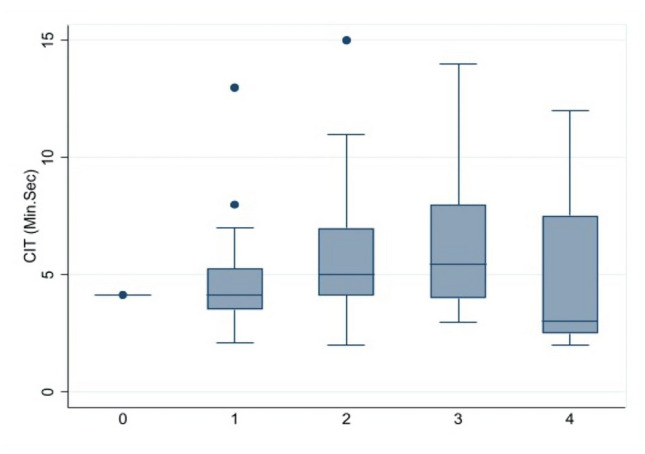
Mean of cecal intubation time among spasm.

**Figure 4:**
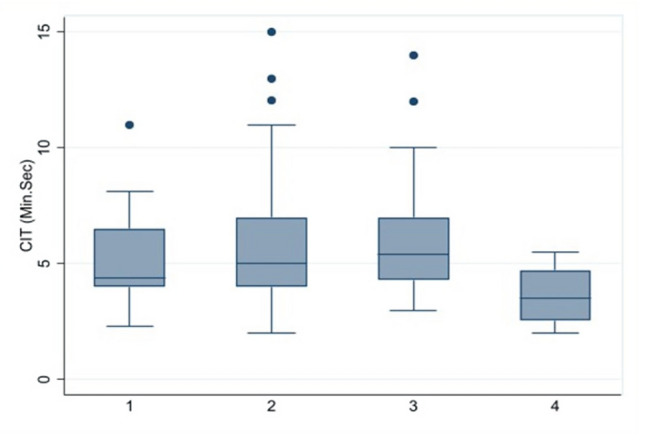
Mean of cecal intubation time among cleanliness.

The efficacy in terms of cecal intubation time or the time from the colonoscopy insertion into the anal verge until reaching the cecal base showed similar with median (range) time about 5 (2, 14) and 5 (2, 15) minutes with no statistically significant in antispasmodic agent group and control group, respectively. All patients had successful colonoscopies and completed discharge without any complications, see Table 2 and Fig. 2. Sub-group analyses were applied for significant variables such as spasm and cleanliness. The cecal intubation time by the spasm was longer in spasm domain at scores 2 and 3 than at scores 0, 1, and 4 but no statistically significant, see Fig. 3. Sub-group of cecal intubation time in terms of cleanliness suggested at scores 4 was better than scores 1, 2, and 3 but no statistically significant, see Fig. 4.

**Table 2:** The scores of outcome among 2 interventions.

Domain	Antispasmodic group (n = 51)	Control group (n = 53)	*p* value
Cecal intubation time (minutes), median (range)	5 (2, 14)	5 (2, 15)	0.953
Pain, mean (SD)	2.0 (0.8)	2.0 (0.7)	0.805
Pain score by VAS, mean (SD)	2.6 (1.4)	3.1 (1.7)	0.098
Spasm, mean (SD)	1.8 (0.8)	2.3 (0.8)	< 0.001
Difficulty, mean (SD)	2.0 (0.9)	2.2 (0.9)	0.198
Cleanliness, mean (SD)	2.4 (0.9)	2.1 (0.7)	0.048

VAS, visual analog scale (0–10).

### Secondary efficacy outcome

The efficacy in terms of satisfaction scores of surgeons with 4 domains including; pain, spasm, difficulty, and cleanliness. Three domains that showed differences were pain, spasm, and cleanliness. The mean satisfaction scores of pain indicated that no difference scores between two intervention groups with no statistical significance. In contrast, in mild pain and severe pain in antispasmodic agent group seem to be higher about 33.3% and 35.3%, respectively, than control group. The pain score using VAS measurement suggested the mean pain score in control group was higher about 3.1 (1.7) than antispasmodic agent group about 2.6 (1.4) but no statistically significant.

In the domain of satisfaction of spasm, the mean scores in control group were higher by about 2.3 (0.8) than antispasmodic agent group by about 1.8 (0.8) with highly statistically significant. Moreover, the domain of satisfaction scores of cleanliness suggested differences between antispasmodic agent group and control groups of 2.4 (0.9) and 2.1 (0.7), respectively, with statistical significance.

In addition, the last domain of satisfaction difficulty scores indicated no difference between antispasmodic agent group of 2.0 (0.9) and control group of 2.2 (0.9) with no statistically significant, see Tables 2 and 3.

**Table 3:** Distribution of scores among 2 interventions.

Domain	Antispasmodic group (n = 51)	Control group (n = 53)	*p* value
Pain, N (%)			0.016
0; no pain	0 (0.0)	0 (0.0)	
1; mild pain	17 (33.3)	10 (18.9)	
2; moderate pain	16 (31.4)	31 (58.5)	
3; severe pain	18 (35.3)	11 (20.7)	
4; very severe pain	0 (0.0)	1 (1.9)	
Spasm, N (%)			0.009
0; no spasm at all	1 (1.9)	0 (0.0)	
1; spasm that did not interfere with colonoscopy insertion	19 (37.2)	9 (16.9)	
2; spasm strong enough to interfere with colonoscopy insertion but not sufficient to prevent completion of the colonoscopy	22 (43.1)	21 (39.6)	
3; spasm severe enough to prevent completion of the colonoscopy and/or to push the colonoscopy backward out of position, and with intermittent air insufflation needed throughout the examination	9 (17.6)	19 (35.8)	
4; spasm severe enough to prevent completion of the colonoscopy and/or to push the colonoscopy backward out of position, and with continual air insufflation needed throughout the examination	0 (0.0)	4 (7.5)	
Difficulty, N (%)			0.242
0; very easy	2 (3.9)	0 (0.0)	
1; easy	15 (29.4)	11 (20.7)	
2; not easy but not difficult	17 (33.3)	24 (45.3)	
3; difficult	16 (31.4)	14 (26.4)	
4; very difficult	1 (1.9)	4 (7.5)	
Cleanliness, N (%)			0.007
0; hard stool, more than 50% of the circumference of colon cannot be visualized	0 (0.0)	0 (0.0)	
1; smooth stool, more than 50% of the circumference of colon cannot be visualized	11 (21.6)	10 (18.9)	
2; smooth stool, between 25 and 50% of the circumference of colon cannot be visualized	12 (23.5)	29 (54.7)	
3; smooth stool, less than 25% of the circumference of colon cannot be visualized	22 (43.1)	12 (22.6)	
4; no stool	6 (11.8)	2 (3.8)	

## Discussion

In this RCTs, the mean of cecal intubation time in this study was no different between antispasmodic agent group and control group. We found that cecal intubation time might be affected by gender, and BMI but was not associated with age, laxative usage drug, abdominal pelvic operation history, cleanliness, spasm, and difficulty. These results are the same as previous studies in the association between gender and BMI with cecal intubation time^13,31–33^. Although we found no difference in cecal intubation time between two interventions, the level of spasm, cleanliness, and difficulty was increased, and the mean of cecal intubation time also increased. The findings suggest that preventing spasms and enduring cleanliness should decrease the difficulty and cecal intubation time. However, in our study, cecal intubation time indicated no significant difference between two groups. A good explanation for these results, the properties of antispasmodic agents that effects to the smooth muscles of human intestines by bloking calcium channels and nervers system, which cause by paralysis of intestines. Therefore, those properties might increase or no different of cecal intubation time^21,34^. In addition, there might affects from the learning curve and the variation of experiences of the physician. Therefore, antispasmodic agents might be helpful and highly efficacy for new experienced physicians or trainees who performed colonoscopy^13^.

Although the usage of antispasmodic agents in our study are similar between the two groups of intervention, the scores in 3 domains i.e., spasm, cleanliness, and difficulty indicated lower in antispasmodic agent than control group. The distribution of the scores showed the same direction as the mean scores of those 3 domains. The contribution of the patients who had more than grade 3 in spasm and difficulty showed about 43% and 34% in control group. In addition, in antispasmodic agent group, about 55% than control group about 26%. In contrast, the satisfaction of pain in our study suggested no difference but the pain score by VAS indicated lower pain scores in antispasmodic agent group than control group. These results confirmed the effects of antispasmodic agents that can relieve the colonoscopy and increase the tolerance of the patients.

From our results, the effect of antispasmodic agents seemed to be better than control group in terms of satisfaction scores of pain, spasm, cleanliness, and difficulty but there could not be a difference in cecal intubation time among two groups. The mechanism of antispasmodic agent/drug that can decrease or relieve the symptom of spasm of the smooth muscle. In the same as our direction, the previous RCT confirmed that the effects of antispasmodic agents remain unclear in terms of short cecal intubation time and the reduction of spasm symptoms. In addition, it was suggested that warm water can also decrease the discomfort at no additional cost^35^.

In contrast, two previous RCTs studied the effect of the antispasmodic agents at the point of decreased symptoms of spasm. Therefore, that symptom might affect the visualization of the scope insertion, cecal intubation time, and pain. However, the results suggested that no beneficial effect from antispasmodic agents^15,16^. Furthermore, another RCT explored the efficacy of atropine from 77 patients with confirmed that no significant benefit of atropine as premedication for colonoscopy procedures^17^.

On the other hand, two RCTs studied the patients who received intravenous antispasmodic medication before colonoscopy. There was a shorter intubation time range from 9.2 to 13 min in antispasmodic group^20,21^. Unfortunately, two RCTs indicated no benefit in both oral and intravenous antispasmodic agents before colonoscopy^18^ and increased insertion time to reach the cecum based^13^.

More recent RCTs, found the efficacy of antispasmodic medication before colonoscopy in terms of lower pain, difficulty, and increased quality of life as same as our results^19,36,37^. In addition, 1 published systematic review and meta-analysis confirmed the benefit of using antispasmodic agents before colonoscopy in decreased irritable bowel syndrome, increased the rate of abdominal pain improvement, improved the rate of relieved abdominal distention/bloating, and without any adverse event^22^.

Our study has several strengths. Firstly, this is the first RCT that confirmed the results of the efficacy of the usage of the antispasmodic agents before colonoscopy. Secondly, this RCT was a strict protocol with enough power to conclude and confirm the results to apply for physicians’ consideration. Thirdly, the loss of follow-up rate and protocol violation did not occur in this study, thus the results were not affected by those reasons. Lastly, our study performed the sub-group analyses to explore the possible association among co-variables with the main outcome.

However, one important limitations could not be avoided. The severity of the disease or the condition of the patients might affect the outcomes, which should be a concern.

## Conclusions

The usage of antispasmodic agents before colonoscopy did not shorten of cecal intubation time. The outcomes of spasm, cleanliness, and difficulty seem to be better than no prior medication. However, further severity of diseases, conditions of the patients, and the steps of bowel preparation with high enough sample size should be further assessed.

## Data Availability

The datasets used and/or analysed during the current study available from the corresponding author on reasonable request.
